# Alcohol epitheliectomy with mechanical debridement in a case of granular corneal dystrophy with r555w homozygous mutation of TGF B1 gene

**DOI:** 10.4103/0301-4738.64126

**Published:** 2010

**Authors:** Prashant Garg, Aneeta Jabbar

**Affiliations:** Cornea and Anterior Segment Service, L V Prasad Eye Institute, L V Prasad Marg, Hyderabad, India

**Keywords:** Alcohol epitheliectomy, granular corneal dystrophy, homozygous mutation of TGF B1 gene

## Abstract

An eight-year-old girl, an offspring of a consanguineous marriage presented with multiple anterior stromal geographic corneal opacities in both eyes. She was diagnosed to have superficial variant of granular dystrophy based on the family history, clinical features and mutation of TGF B1 gene. She was treated by alcohol-assisted removal of epithelium followed by mechanical debridement of abnormal deposits. Postoperatively, the cornea in both eyes was clear with no trace of opacity and the patient had an unaided visual acuity of 20/20 partial.

The classical granular corneal dystrophy, an autosomal dominant disorder of TGF B1 gene is characterized by sharply demarcated breadcrumb-like deposits.[1,2] The patients usually present in the fourth decade of life and the disease progresses very slowly. However, patients who are homozygous for the gene present in the first decade of life and the disease is characterized by deposits predominantly located in the anterior stroma / Bowman's plane. These deposits have a high tendency to recur necessitating repeated surgery.[3‐6] These facts are important considerations in the management of this disorder. We describe a new surgical option for this disorder.

## Case Report

**Figure 1:**
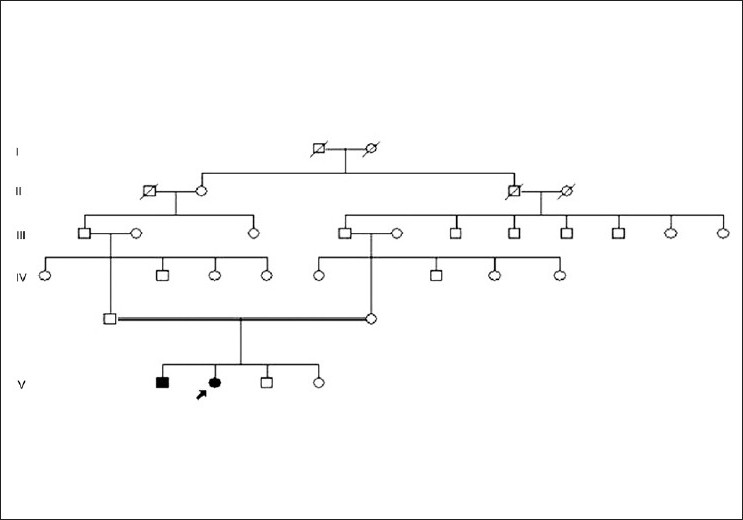
Pedigree chart of the family. The proband is shown with the arrow. The parents of the proband are cousins

An eight-year-old girl presented to us with complaints of blurring of vision and episodes of ocular irritation in both eyes since the past four years. Her nine-year-old brother also had similar complaints. Both children were offsprings of a consanguineous marriage and the family pedigree is shown in Fig. 1.

**Figure 2:**
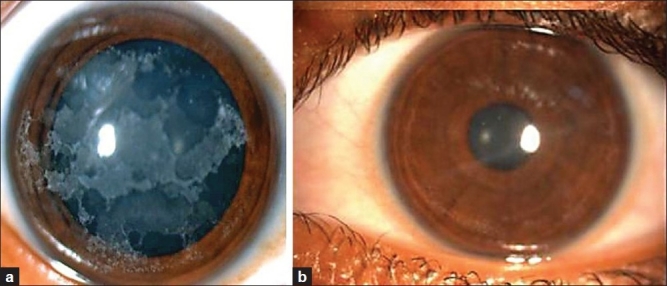
Clinical picture of the right eye of the patient showing diffuse superficial opacities (a). Same eye after the surgery (b)

**Figure 3:**
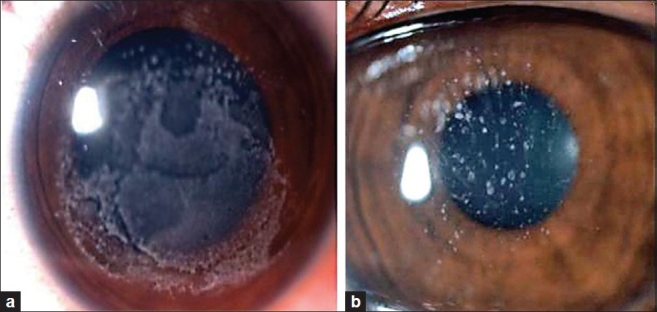
Clinical picture of the right cornea of the brother (a) and father (b) under diffuse illumination

On examination her visual acuity was 20/50 with -0.75 diopter cylinder (D Cyl) × 90° in the right eye and 20/50 with -1.00 D Cyl × 90° in the left eye. Slit-lamp examination showed multiple anterior stromal geographic corneal opacities in both eyes [Fig. 2a]. The rest of the anterior and posterior segment examination was essentially within normal limits. Her brother had identical corneal findings [Fig. 3a]. Both parents had classical granular dystrophy [Fig. 3b]. Genetic analysis of family members showed the presence of Arg555Trp mutation of TGF B1 gene. The mutation was homozygous in the children.

The patient underwent alcohol epitheliectomy with mechanical debridment under general anesthesia. An 8-mm trephine was placed on the cornea and 2 ml of 20% ethyl alcohol poured in the well of the trephine was allowed to stay for 60 sec. The alcohol was thoroughly washed with saline. The epithelium was removed with a dry Q-tip applicator. Underlying deposits were then scraped off with a No. 15 surgical blade. The deposits came off easily leaving behind clear corneal stroma. At the conclusion a bandage contact lens was placed.

Postoperatively she was given prednisolone acetate eye drops four times per day and ofloxacin eye drops four times per day. After one week the bandage contact lens was removed; ofloxacin was discontinued and the prednisolone acetate was tapered. The procedure was repeated for her left eye after four weeks. At the end of six weeks follow-up after the second eye surgery the patient's unaided visual acuity improved to 20/20 partial in both eyes. Cornea was clear except for mild anterior stromal haze on slit-lamp examination [Fig. 2b].

## Discussion

Homozygous granular corneal dystrophy poses a therapeutic challenge. Various treatment options for this condition are superficial keratectomy, photo therapeutic keratectomy (PTK), lamellar keratoplasty, and penetrating keratoplasty.[3‐5] PTK has the following drawbacks: need for an access to excimer laser, expense, difficulty of general anesthesia in the laser room especially for treating young children, thinning and consequent hyperopic shift. Therefore, PTK might not be the best option in managing this condition. Penetrating keratoplasty and lamellar keratoplasty are challenging in children due to the need for repeated examinations under anesthesia, an unpredictable refractive outcome with amblyogenic potential, and increased risk of rejection. These disadvantages have a major bearing on the treatment of the disorder due to a high potential for recurrence.

In contrast the treatment described in this paper is safe for children, does not alter corneal thickness and refraction and can be easily repeated. We got the clue for this new treatment from the observation that the accumulation of the abnormal material is restricted to the Bowman's layer and removal of the corneal epithelium would expose the deposits for mechanical debridement.
